# Artificial Intelligence and Machine Learning Based Intervention in Medical Infrastructure: A Review and Future Trends

**DOI:** 10.3390/healthcare11020207

**Published:** 2023-01-10

**Authors:** Kamlesh Kumar, Prince Kumar, Dipankar Deb, Mihaela-Ligia Unguresan, Vlad Muresan

**Affiliations:** 1 Department of Electrical and Computer Science Engineering, Institute of Infrastructure Technology Research And Management, Ahmedabad 380026, India; 2 Department of Chemistry, Technical University of Cluj-Napoca, 400114 Cluj-Napoca, Romania; 3 Department of Automation, Technical University of Cluj-Napoca, 400114 Cluj-Napoca, Romania

**Keywords:** medical infrastructure, healthcare infrastructure, artificial intelligence, artificial intelligence in healthcare, deep learning, machine learning, healthcare and AI

## Abstract

People in the life sciences who work with Artificial Intelligence (AI) and Machine Learning (ML) are under increased pressure to develop algorithms faster than ever. The possibility of revealing innovative insights and speeding breakthroughs lies in using large datasets integrated on several levels. However, even if there is more data at our disposal than ever, only a meager portion is being filtered, interpreted, integrated, and analyzed. The subject of this technology is the study of how computers may learn from data and imitate human mental processes. Both an increase in the learning capacity and the provision of a decision support system at a size that is redefining the future of healthcare are enabled by AI and ML. This article offers a survey of the uses of AI and ML in the healthcare industry, with a particular emphasis on clinical, developmental, administrative, and global health implementations to support the healthcare infrastructure as a whole, along with the impact and expectations of each component of healthcare. Additionally, possible future trends and scopes of the utilization of this technology in medical infrastructure have also been discussed.

## 1. Introduction

**Figure 1:**
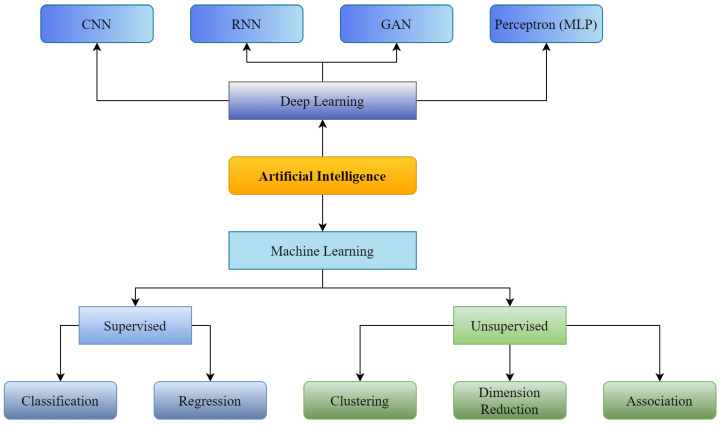
Supervised and Unsupervised machine learning with Convolutional Neural Network (CNN), Recurrent Neural Neural Network (RNN), Generative Adversarial Network (GAN) as branches of Deep Learning.

Artificial Intelligence (shown in Figure 1) was initially introduced in the medical sector in 1976 when a computer algorithm was used to determine the reasons for intense abdominal pain [1]. From the first healthcare implementation of AI to today, numerous applications of AI have been introduced to enhance the strength and overcome the shortcomings of available medical infrastructure. These implementations include assistance in disease detection, like diabetes detection or cancer detection; enhancement of pathology classification, such as classification of radiology scans and outlining electrocardiogram qualities for cardiac study [2]; and forecasting illnesses with algorithms based on Machine Learning (ML) and Deep Learning (DL) developed to solve problems such as the pandemic of COVID-19 [3,4], serving as an epitome. However, despite the healthcare industry’s considerable investment in technological advancements, its deployment and integration in healthcare are still in their preliminary stages [5]. Workforce scarcity and exhaustion, and the transition to long-term illness care, are among the most significant concerns in healthcare. Thus, AI can significantly enhance the healthcare infrastructure through its extensive applicability.

AI is revolutionizing medical infrastructure significantly in diagnosing various diseases, using medical imaging from various available medical imaging formats like- X-rays, MRI, CT, etc. AI can easily detect diseases related to the skin, lungs, organs, and viral issues. For instance, some skin diseases include skin cancer, acne, and rashes. Early identification of such skin illnesses can prevent critical future problems. Furthermore, in this direction, researchers like- Shoieb et al. [6] classified skin cancer using available data consisting of cancer images. Their results showed a considerable increase in skin diagnostic accuracy and precision compared to earlier studies. Zaher et al. [7] and Charan et al. [8] presented such a model to detect breast cancer using radiology scans. Moreover, like skin and breast cancers, lung cancer is amongst the deadliest ailments across the world [9,10] that causes 7.6 million yearly deaths worldwide [11]. Moreover, early detection of such a deadly disease is the only possible cure to reduce this number [12]. Many researchers [13,14,15,16,17] have proposed AI and ML-based approaches for predicting lung cancer using various sources. Apart from these applications, researchers have used AI for the detection of tumor [18], tuberculosis [19], and even COVID-19 diagnosis [20] as well, mainly using chest X-rays. Medical imaging for disease diagnosis and prognosis is widely accepted and increasing with boundless expectations and improvements in conventional medical infrastructure.

The imaging data is machine-readable, allowing the ML and DL algorithms to be run after adequate preprocessing or quality check steps. Moreover, a substantial chunk of healthcare data, including clinical laboratory reports, physical examinations, discharge summaries, and operation notes, usually remains narrative, which would be amorphous and inaccessible to computer algorithms. In this situation, Natural Language Processing (NLP) aims to gather relevant data from the available chunk to support clinical judgments [21]. Based on existing records, NLP uses text processing to define disease-related phrases in medical documentation [22]. Subsequently, keywords are selected after assessing their influence on categorizing normal and abnormal instances. For example, Miller et al. [23] employed NLP to track undesirable events in the laboratory environment. In addition, NLP pipelines can aid in illness detection. This technology has also been used for detecting various disease-related factors for cerebral aneurysms using clinical notes [24] to distinguish normal individuals from patients suffering from cerebral issues.

Moreover, Afzal et al. [22] used NLP to extract peripheral arterial disease-related keywords from clinical narratives. These were then utilized for differentiation between peripheral arterial disease and normal patients. Not only to collect documentation about disease-related information but NLP is being explored to learn various suicide factors [25] from suicide notes by developing a vocabulary or language-specific database. Moreover, this branch of Artificial Intelligence is utilized for evaluating mental illness [26], understanding the clinical workflow [27,28], classifying medical prescriptions [29], forecasting patient predilection [30,31], predicting risk and stratification of a patient [32], making decision support system [33], and question answering [34]. Juhn et al. [35] have also introduced an autonomous system that can significantly reduce the burden of medical triage by collecting patient data and understanding it with NLP to help the patient while choosing a consultant and completing other procedures, which usually take a long time in any hospital building.

Robotics focuses on designing and developing robots. When combined with AI, the result is an intelligent machine that can be taught to undertake complicated processes requiring much thought and continual learning. Consequently, a further branch of AI is interested in educating a robot to interpret the world in predicated but generic ways, control things in intractable surroundings, and communicate with humans. Robots that may undertake complex surgical treatments, such as minimally invasive and surgeon-less surgeries, are known as “Surgical Robots”. The systems represented [36,37] are the gold standard of care in many laparoscopic operations, with approximately a million operations performed each year. Robotic surgery enhances the effectiveness, precision, and reliability of surgical operations allowing quicker recovery and better patient outcomes. Apart from surgical tasks, in healthcare, there are several duties related to management. The application of AI in this domain has less adaptability than acute services, but it can deliver substantial productivity. It is necessary for hospitals because, for instance, a US nurse spends an average of 25% of her job tenure on administrative tasks [38]. This aim is most likely connected to robotic process automation technology. It is used in various medical systems, such as user registration, medical documentation, payment flow administration, and clinical record-keeping [39,40]. Besides patient interactions, mental well-being, telemedicine, and chatbots are often used in other medical contexts.

Research and development are some of the most critical areas, and boosting these areas can significantly strengthen healthcare infrastructure. For example, machine (and deep) learning algorithms have been used in a variety of drug discovery processes, including physio-chemical, poly-pharmacology, drug repositioning, quantitative structure-activity relationship, pharmacophore modeling, drug monitoring and revealing, toxicity prediction, ligand-based virtual screening, structure-based virtual screening, and peptide synthesis activities [41]. In addition, pharmacogenetics and molecular biomarker technologies may forecast drug efficacy and medication reactions within subjects, essential to precision medicine progress [42].

A significant number of studies [43,44] conducted in revolutionizing the conventional drug design include DeepMind at Google and AlphaFold, a tool based on AI, trained on protein binding domain spatial information to estimate the multi-dimensional shape of a protein from the sequence of amino acids. AI has become an effective tool in today’s technology because it saves time and money. Such rapid discovery and development of drugs can save millions of lives in critical conditions like a pandemic, which can be defined as an explicit strengthening of overall infrastructure by reducing overall development costs with increased drug efficacy [45,46,47].

Furthermore, supplying incorrect dosage is one of the hackneyed issues in this sector that not only causes the loss of millions of dollars but also weakens the whole infrastructure by increasing the mortality rate with undesired and deadly side effects [48]. With the rise of AI, numerous scientists are turning to ML and DL techniques to identify optimal medicine dosages. For example, Shen et al. [49] created an AI-based system called AI-PRS to discover the best medication doses and combinations for HIV treatment using antiretroviral therapy. Julkunen et al. [50] also created comboFM, a unique ML-based tool for determining optimal medication coalescing and dosing in pre-clinical investigations such as cancer cells. CombinationFM uses factorization machines—a machine learning framework to analyze multi-dimensional data and discover optimum medicine combinations and doses. Xue et al. [51] have also identified a suitable bioactive agent and inspected the drug delivery.

As discussed above, AI has become an expert in many stages of drug distribution and optimization. Studies also show how AI can further help in rapid discoveries and development of drugs by working on various stages like predicting interactions between proteins and their foldings [52], ligand and structure-base virtual screening [53,54], quantitative structure-activity relationship modeling and drug re-purposing [55,56], estimating physicochemical properties and bioactivity [57,58], toxicity and mode of action prediction of the compound [59,60], recognition of molecular pathways polypharmacology [61,62], de novo drug designing [63], pharmaceutical manufacturing and clinical trial design [64], and related ones [65,66], to various crucial, even may be incurable, diseases with its unbounded intelligence and memory power on 0.9-micron thick silicon bridges, also known as memory chips. All these studies show how AI enhances drug research and development for strengthening medical infrastructure economically and in terms of rapid processing.

This study evaluates advancements in artificial intelligence and machine learning to strengthen medical infrastructure. We are presenting a comprehensive study on the most widely used implementations of Artificial Intelligence, with its branches of Deep Learning and Machine Learning, while analyzing their impacts on the healthcare infrastructure. The existing reviews do not provide a comprehensive analysis, including the closest possible impact of recent advancements in costs, patient-service satisfaction, healthcare staff efficiency, operation time, etc. In contrast, this article draws an overview by studying various case studies, including the employment of AI and ML-based solutions to solve one or more problems in the medical sector and their results. We also analyze how the advanced use of human cognitive skills in machines could change medical infrastructure in the future.

A literature search of articles published between May 2015 and 30 November 2022, was conducted by PRISMA guidelines [67] using the search terms “Artificial Intelligence (AI)”, “Machine Learning (ML)”, “Deep Learning (DL)”, “AI in Healthcare”, “ML in Healthcare”, “DL in Healthcare”, “AI in medical infrastructure”, “ML in medical infrastructure”, “DL in medical infrastructure”, “Healthcare technologies”, and “Smart healthcare infrastructure” on IEEE Explorer, Web of Science, ScienceDirect, PubMed, and arXiv electronic databases. Non-English and duplicate articles were excluded from the initial 135 research papers found in the first round of search; a total of 45 articles remained. Full texts of all these articles were acquired. After a thorough analysis and exclusion based on strict criteria of utilizing AI and ML at an infrastructural level, 25 papers are used to write this review.

## 2. Evolution of AI and ML in Medical Infrastructure

**Figure 2:**
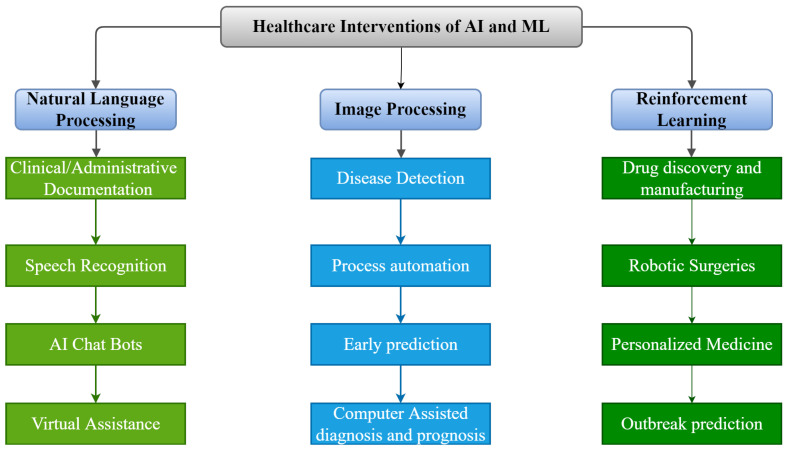
Applications of AI and ML in Medical Infrastructure.

As shown in Figure 2, there are numerous possible applications of AI and ML algorithms to develop efficient tools to strengthen the healthcare infrastructure. Over the last five decades, AI has dramatically impacted the medical infrastructure. The scope of AI and ML applications has increased, opening the doors to individualized medicine rather than algorithm-based treatment. Predictive models that predict illness, treatment responses, and even preventive medicine in the future may be developed using such models [68]. AI may strengthen the healthcare infrastructure by improving diagnostic accuracy, clinical operations and workflow, procedure accuracy, treatment monitoring, and overall patient satisfaction. The evolution of AI and ML in medicine is detailed in the following timeline.

The late nineteen seventies were driven by a perceived limit of AI, which increased till the early 1970s, driven by high expenses in establishing and sustaining an expert database of information in digital forms. Nevertheless, while reluctantly drawing public attention, many researchers continued their studies in this field with mutual collaborations. As a result, in 1971, Saul Amarel of Rutgers University began working on his study on applications of computers in bio-medicine. In 1973, Stanford University also made significant efforts to enhance communication strength among many universities to focus on clinical and biomedical studies [69]. Rutgers University also funded an AI workshop in 1975 to spread awareness about its applications towards strengthening medical infrastructure [70].

Further, a causal-associational network [71] was used to construct a glaucoma consultation tool, one of the prototypes to show that AI might be used in medicine. This system comprises model development, a database, and consultation. This model can apply certain illness information to individual patients and provide treatment suggestions. Another system called MYCIN, an AI system that uses “backward chaining” was created in the early 1970s [72]. Physicians may enter patient information into MYCIN to get a list of probable infections for the system to offer antibiotic treatment alternatives tailored to each patient’s weight.

The University of Massachusetts launched DXplain in 1986 as a decision support system. This software generates a list of diagnoses based on a patient’s symptoms. An electronic medical textbook provides extensive explanations of illnesses and links to supplementary resources. It could offer an analysis of 500 different diseases with its early versions. Later on expanded to more than 2400 disorders [73], which can help the healthcare sector to collect important information about many crucial diseases from a single place that helps to escalate many processes of research and developments. At the end of the 1990s, accelerated beliefs in machine learning capabilities, notably in the medical field, helped pave the way for the contemporary era of AI in healthcare infrastructure, coinciding with the aforementioned technical advancements.

Another system, similar to DXplain, Watson, was built by IBM in 2007, a question-answering open-source system that earned first place on Jeopardy’s television show in 2011. Unlike traditional systems, using reasoning in forward-backward methodologies or hand-crafted rules, various searches along with NLP were used to analyze unstructured content and find probable answers [74]. Using this method was more convenient, and it was also less expensive and simpler to maintain.

Medical records of patients, including other electronic resources, helped to use technologies like DeepQA to provide professional medical suggestions and related information. It provided new opportunities for making therapeutic decisions based on evidence [75]. The binding of RNA proteins was effectively identified by Bakkar et al. [76] using IBM Watson in 2017. Digitized medicine became more accessible due to this impetus, combined with enhanced computer hardware and software applications. Natural Language Processing has also revolutionized chatbots by allowing them to engage in meaningful conversations. In 2011, a virtual assistant known as Apple’s Siri used this technique. Amazon also used a similar technique for its virtual assistant, called Alexa. Pharmabot and Mandy are chatbots established in 2015 and 2017 to help young patients and their parents better understand their medications [77,78].

**Figure 3:**
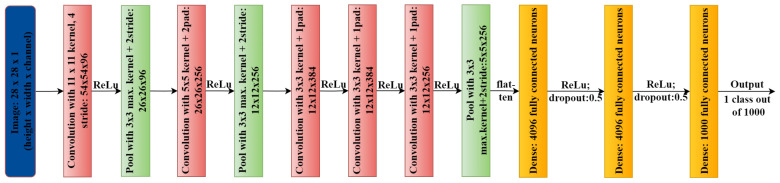
AlexNet architecture.

In image processing, convolutional neural networks (CNNs) are widely used for feature detection and learning. To develop specialized filters, CNN uses several layers that evaluate an image and look for certain patterns. Several CNN algorithms, such as Le-NET [79], AlexNet [80] (shown in Figure 3), VGG [81], GoogLeNet [82], and ResNet [83], are now readily accessible. Such models are useful for medical image analysis and working in many other domains to strengthen the overall infrastructure by entitling every component of the healthcare sector. For example, MetaAI is one of the major research organizations working in the direction of utilizing AI and ML algorithms for more generalized purposes, like Computer Vision, Conversational AI, Integrity, Natural Language Processing, Ranking and Recommendations, Systems Research, Speech and Audio, Robotics, and Graphics- MetaAI and other big tech companies, like Google, Amazon, and Microsoft, work in these fields with enormous resources. One can find their research in various healthcare domains, for example, Google’s research on disease detection in eyes using external photographs [84], and Microsoft’s research in the biomedical natural language field processing [85]. These advanced researches are dedicated to healthcare because they validate million-size datasets and their diversity.

The analysis presented above shows the significant development in AI and ML-related research and its active utilization in healthcare infrastructure and its enhancements in every possible manner, with the employment of such advanced technologies that enable machines to think intelligently. Therefore, AI and ML’s role is to empower medical infrastructure from its roots by following or enhancing the fundamental requirements. Furthermore, the involvement of AI and ML ensures precision and safety simultaneously without breaking any ethical substance.

## 3. ML and AI Based System for Quantitative Analysis

AI and ML applications can help sustain the medical infrastructure by playing crucial roles in its minute components to bolster the more considerable structure while improving from the ground. Moreover, this can only be done if this technology solves fundamental problems, such as reducing diagnostic time, hospital stays, and complications while allowing doctors to perform finer operations to strengthen the infrastructure. However, these advantages must be weighed against the longer intra-operative time-frames, higher financial expenditures, and more significant training load associated with AI-powered procedures, which should also be considered carefully. Although the results of such a cost-benefit analysis are only sometimes, only sometimes consistent, almost every portion of healthcare infrastructure provides significant support for their usage and further improvements. Such affirmative impacts can be supported by several case studies, as follows:

### 3.1. AI Supported Robotic Surgeries

While consuming any healthcare service, operations or surgeries are the most expensive part that any patient suffers. Moreover, the cost of such surgeries is usually high because they need individual specialists and detailed analysis of each critical detail. After all, even a trivial tolerance can cause a life-or-death situation. Therefore, the plantation of machine intelligence that can provide solutions without a single chance of error would not only help to decrease the overall cost of such operations and surgeries but also help the hospitals to reduce the queue size while attending more and more individuals every single day, which would eventually result in a win-win situation for both patients and hospitals. One possible solution that can work in the direction of the field mentioned above of interest is AI-supported robotic surgeries. For example, intra-corporeal suturing (using suture to stitch up an incision or wound), often used in urological and gynecological operations, may be improved with the use of robots [86].

#### 3.1.1. Urological Applications

In the United States, radiation prostatectomy (a procedure to remove an organ or gland using radiation therapy) has been projected to be performed with robot assistance regularly. Urology has been the specialty most eager to embrace robotic surgery [87], beginning from robot-assisted laparoscopic (medical process to treat internal body organs/parts without large incisions) prostatectomy (RALP). Because of this, some studies have compared it to open and laparoscopic radical prostate surgery (LRP). For example, Tewari et al. [88] presented a comprehensive study on 62389 RALP, 57303 LRP, and 167184 open surgeries while monitoring prostate-specific antigen levels, pathological features, and age. According to their analysis, Table 1 summarises that RALP surgeries are better than open and LRP surgeries in terms of the reduced amount of blood loss and hospital stay.

There were also reduced rates of readmission, nerve damage, and re-operations in RALP compared to LRP, as well as higher rates of continence and the recovery of sexual function [89,90] in RALP compared to LRP. In a further study, Sukumar et al. [91] analyzed the oncological outcomes of 5152 patients who had RALP. A high-volume tertiary center showed that RALP’s long-term biochemical control was successful. These studies imply that RALPs are usually more effective and cost-efficient because of their lower side-effect tendencies and low chances of repetition requirements. These results can help to understand the efficiency of healthcare services from both a patient and hospital perspective.

Furthermore, researchers from European countries have also done several studies in the direction of the utilization of robotic surgery and found significant applications for this technology, particularly for RALP. A decade ago, open and laparoscopic procedures had comparable, if not better, results than RALP. Research by Hakimi et al. [92] has suggested an explanation for this, considering the brief period in which robotic surgery has been accessible. It was discovered that in a case series of more than 300, the initial seventy-five RALP surgeries out-performed the final seventy-five LRP cases, illustrating the shorter learning curve of the robot vs. the conventional laparoscopic method [93]. As a result of this, and the rising amount of evidence supporting the effectiveness and safety of RALP, robotic surgery for prostate cancer is likely to become the procedure of choice shortly.

#### 3.1.2. Gynaecological Interventions

Endometrial (lining of a woman’s hollow, muscular uterus, which is located in her pelvis) and cervical cancer patients may now undergo radical hysterectomy (uterus removal process) using a robot-assisted surgical technique that the Food and Drug Administration first licensed in 2002 for use in urology [94]. After that, robot-assisted surgery became a common practice in the USA. However, despite no randomized controlled trials, the second most common robot-assisted surgery in the United States has not been studied in the same way as RALP.

Kruijdenberg et al. [95] looked at data from 342 hysterectomy patients and 914 total laparoscopic radical hysterectomy patients in individual case series. Even though patients undergoing robot-assisted radical hysterectomy required fewer blood transfusions than those undergoing total laparoscopic radical hysterectomy (9.7% vs. 5.4%) and had shorter lengths of stay, severe complications could occur after surgery were more common in robot-assisted radical hysterectomy patients. However, they did determine that trials for this method were smaller in population and that surgical device experience after the initial learning phase can further mitigate the complexity rate, reducing the cost of the service consumption for the patient and increasing the hospital’s availability to attend to more patients. However, there are some drawbacks to such surgeries in the form of possible complications after the operation.

#### 3.1.3. Cost Analysis

Considering the potential advantages of robotic surgery, such as shorter hospital stays and fewer complications, we should also consider the financial support required to make it possible for a robot to operate under the guidance of an AI and a human expert, requiring the medical personnel to undergo technical training to cope with a machine and perform crucial tasks while taking care of innocent lives.

The employment of robots in gastrointestinal, thoracic, urological, and gynecological surgery is beneficial [96]. For instance, comparing laparoscopic and robot-assisted radical prostatectomy for a controlled but randomized study, it was found that urine erectile dysfunction, incontinence, and readmission rates were decreased in a group of subjects when diagnosed one-year post surgery [97,98]. Robot-assisted laparoscopic bypass costs lesser than its gastric version; however, new research assessed the whole expenses of each treatment, including the complications costs, patient-stay time, and explicit process costs [99,100]. According to these studies, laparoscopic and open surgeries cost $2334 and $3637 more than robot-assisted surgeries, respectively. The results also encourage the wide use of robot-assisted surgeries, instead of conventional methodologies, to observe this cost-reduction phenomenon at an infrastructural level. In addition, a unit of surgical robots might cost anywhere from $1 million to $2.5 million to set up [101]. Robot-assisted procedures cost around $2000 more each operation than the identical treatment done with traditional laparoscopy, with annual maintenance expenses estimated at $138,000 per year [102].

Robotic surgery may be cost-effective, but the initial expenditures are exorbitant for many hospitals, making it difficult for small hospitals and healthcare units to utilize this technology at the purchase stage. In addition, surgeons may need more expertise with the procedures related to surgical robots and their operations, and there are a few training facilities equipped with the requisite equipment, which results in fewer options for surgeons to get training [103]. Although due to a dearth of research that provides an itemized breakdown of expenditures for robotic surgery, it seems complicated to evaluate every minute cost efficiency [104], these studies most closely imply that robot-assisted surgeries are cost-effective in terms of overall operational cost for both patients and hospitals. However, the cost of setting up the ground infrastructure for this purpose is relatively high, and many hospitals or individual healthcare units may need more resources.

### 3.2. Tuberculosis Monitoring with AI

A cohort Markov transition state model was made by Salcedo et al. [105] to compare the treatment completion rates and costs of traditional in-person methods called Directly Observed Therapy (DOT) and AiCure for active Tuberculosis (TB) patients consuming treatment in Los Angeles, USA. This model kept track of how TB patients did every month for 16 months, which was the most prolonged period for treatment they saw in the available data. For each sequential month, subjects with TB either continued getting treatment, stopped getting treatment, or got cured. These probabilities were based on specific health department monitoring data on active TB subjects at a public health clinic in Los Angeles. It was assumed that both DOT and AiCure patients had followed a standard treatment plan with an average of 11 doses per month. This average showed that both daily and twice-a-week treatment plans for TB were used depending on what the patient and provider wanted. They mentioned that patients who did not get better with AiCure should be switched back to DOT, which usually happens in any trial, and patients who did not get better with DOT were lost to follow-up. A patient in the study group had to have a confirmed diagnosis and not multidrug resistance or HIV. The health department and the University of Southern California review board reviewed the primary case study with AiCure on people diagnosed with TB.

According to the data of the subjects of the specified region (as shown in Table 2), AiCure proved its usefulness for lowering patient-nurse workloads while attaining comparable success in DOT stages. Patients receiving AiCure finished their therapy at a rate statistically equivalent to that of DOT while spending, on average, $2226 less. The findings of the sensitivity analysis demonstrated that AiCure could be the most cost-effective option for more than 95% of the instances with willingness-to-pay scales more than $50,000 for each quality-adjusted life year, notwithstanding the uncertainty around completion time-frames resulting from the small number of subjects in the data of the region. Even in the cases when DOT was more affordable than AiCure, the study directs towards insignificant inputs (per-patient saved $3672) in comparison with the most considerable reduction of AiCure with significant inputs (per-patient $7603 saved).

Even with a few minor limitations, this study assures the effectiveness of an artificial intelligence-supported tool in terms of cost without lowering the quality of care, as long as it is only used for simple cases of pulmonary TB that are not resistant to drugs. Future research should look into how results differ depending on the patient population and the challenges of implementing such AI-assisted disease monitoring systems on a large scale. Utilizing such tools in the healthcare infrastructure can help reduce the cost of monitoring operations and the workload of a medical professional, which would help the medical staff work more efficiently while attending to more complex cases where human intervention is mandatory.

### 3.3. Polyp Detection and Treatment with AI

This research [106] is a follow-up to a clinical experiment that tested AI’s ability to distinguish between colorectal polyps (noncancerous tumors) and normal tissues. Subjects with a rectosigmoid (type of colorectal polyp) polyp less than 5 mm in diameter were included in the study. A diagnosis-and-leave (DAL) method assisted by AI prediction and a polyp resection (PLR) method were compared in terms of average colonoscopy costs. Here, the rectosigmoid type of predicted polyps is assumed to not be completely visible to AI but cannot be removed. However, another method can detect and remove all types of polyps. Based on the number of colonoscopy reimbursements performed over public health insurance plans for four countries, this study computed the gross yearly expenditures of colonoscopies.

**Figure 4:**
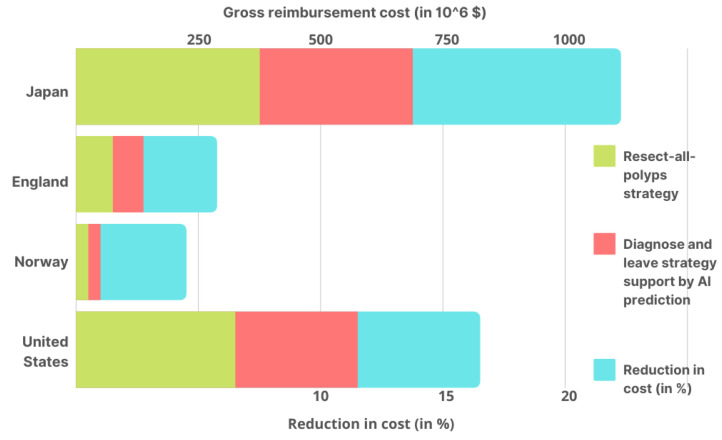
Using the DAL method assisted by AI prediction and the PLR strategy in Japan, the United States, Norway, and England, the estimated gross yearly reimbursement for colonoscopies in each country’s AI cost reduction % is also given.

Under the two techniques, average colonoscopy costs and gross yearly reimbursement were calculated (as shown in Figure 4) for four nations. Taking into account the implementation costs of the AI tool, in Japan, the average cost of colonoscopy under the DAL method was $119 cheaper than the PLR strategy. As a result, colonoscopies are estimated to save $149,220,000 yearly in gross yearly reimbursement savings. According to the National Health Service, a DAL colonoscopy was 52 dollars less expensive than the PLR approach in England. As a result, colonoscopies are estimated to save $12,360,348 per year in gross yearly reimbursement savings. In Norway, it costs $34 less than those in which all polyps are removed, with a saving of $1,114,733 in gross yearly reimbursement savings. The study also found the cost to be $125 less than the PLR strategy for a colonoscopy in the United States, based on Medicare reimbursement rates, with a gross yearly reimbursement savings of $85,244,220 per year.

This study shows how AI can help to lower gross yearly reimbursement for colonoscopies by up to 18.9% in Japan, 10.9%, 7.6%, and 6.9% in the United States, Norway, and England, respectively (Figure 4). These reductions can significantly affect the overall infrastructural cost in any country, which directly strengthens healthcare by lowering expenditures. Moreover, the combined efforts of Artificial Intelligence and DAL colonoscopy can significantly reduce the above-discussed costs with some variations based on the region where these implementations have been successfully employed.

### 3.4. Sepsis Severity Prediction Using ML

In the study [107], ML-based algorithms were implemented and analyzed to predict sepsis severity. The method developed during this research employs just six vital signs to achieve a greater level of sensitivity and specificity than other sepsis scoring systems utilized in hospitals. Patients in intensive care units (ICUs), a critical component of any hospital and equally important in the overall healthcare infrastructure, were the focus of this investigation.

From December 2016 to February 2017, a clinical study was performed at San Francisco Medical Center to evaluate the results of the average duration of stay and the in-hospital death rate. This open-label factorial investigation was accessible to participants ages 18 and older who were admitted to participating facilities. A random allocation process was used to place all enrolled patients in one of the two study fields. Machine learning algorithms (MLA) were applied in the experimental group and the existing severe sepsis detector. When the alarm was received, the medical team assessed the subjects and, if necessary, started treatment related to severe sepsis. Patients who got MLA warnings had their group assignments immediately exposed, even though they were randomly allocated.

A total of one hundred and forty-two individuals were evaluated for trial qualification throughout the study. A halt to patient enrollment occurred before the 150-patient maximum anticipated since the research was completed earlier than expected. However, they all satisfied the inclusion criterion, as none of them were under the age of 18. All the patients were randomly allocated. Seventy-five joined the control group, and the remaining were instructed to join the experimental group. Patient follow-up was seamless because patients were monitored for the whole period of their stay in the hospital, and no further interventions or questions were required. The study came to an end after analyzing data from all subjects.

Clinical and demographic information for all subjects was gathered. The study’s average patient age was 59 years, with women making up 53.5% of the participants and males accounting for 46.5%. Both trails were statistically almost similar in terms of their demographic differences. The medical-surgical high acuity ward, the transfer center, and the emergency room were the three most popular entry points for patients in both study groups. The primary result of this study implies that the hospital length of stay was decreased by 20.6 percent in the experimental group (Table 3). In the group other than experimental, 16 out of 75 patients died while in the hospital, but in the experimental group, 6 of the 67 patients died while in the hospital (as shown in Table 3). The difference in in-hospital mortality rates decreased significantly. There was also a statistically significant reduction in the risk ratio of in-hospital mortality. As a result, the experimental group’s mortality rate dropped by 12.34% compared to another group.

This medical study showed that an ML prediction system for sepsis severity detection improved overall patient outcomes. Compared to the existing rules-based sepsis detector, this method reduced the length of total hospital stay and death by a significant margin. From these findings, ML systems can help to reduce the chances of having severe sepsis by early detection with improved patient outcomes. One of its disadvantages is that it is only applicable to ICUs, and future studies can be focused on to solve this issue. This study also implies the scope of such ML-based prediction systems for the early detection of many diseases, which can significantly decrease the mortality rate and preclude many complexities.

Each of the studies mentioned above is mainly directed toward the pros and cons of utilizing Artificial Intelligence and Machine Learning-based solutions to enhance the individual sectors of the overall medical infrastructure. Still, these studies imply significant possibilities and outcomes of utilizing such advanced technology in healthcare infrastructure and its applicability on a wide scale. These studies provide important implications for the cost-effectiveness of AI and ML-based support in healthcare not only from the patient side in terms of overall service consumption cost but also from the perspective of infrastructure strengthening by the employment of this technology, in terms of reduced mortality rates, reduced operational cost and time, attending more patients for better healthcare distribution, equal distribution of healthcare services, and the cost of applying such technology to the current healthcare infrastructure while utilizing the available technologies to minimize the initial cost of employment.

## 4. Discussions: Future of Medical Infrastructure with AI

The above analysis helps one understand the role of Artificial Intelligence and Machine Learning in automating various essential components and processes on which medical infrastructure is based and cannot be sustained without them. For example, we find how Deep Learning could accelerate the diagnosis procedure; even early detection can preclude many losses; how Natural Language Processing can automate the hospital management processes while saving a lot of time and resources at the same time, and how AI can accomplish complex calculations and estimations with minimal time and resource requirements to find every possible methodology to mitigate the effect of an incurable ailment by predicting its root cause and related antigen to dissolve it. We also discussed the economic aspects of utilizing Artificial Intelligence and Machine Learning based solutions from a hospital and patient perspective. We saw that a significant improvement could be made, especially in long-run implementations, based on the advantages and limitations presented in Table 4.

At the research and development stage, identifying stones from ultrasound images and computed tomography gets praise. Researchers utilized these images and applied various models for detection like CNN, Discriminant analysis, Artificial neural network (ANN), Bayesian model-Decision Tree, and Multi-parametric algorithm. Kazemi et al. [119] applied the Ensemble model and achieved 97.1% of accuracy. The ensemble model indicates the combination of various models; for example, in this study, the Bayesian model–Decision Trees, ANN, rule–based classifiers, are combined for classification. Most studies utilized support vector machines, a method of supervised classification [120]. Artificial Neural network has impacted the diagnosis of Tuberculosis far better than conventional ones. Since tuberculosis data is mainly in X-rays, CNN is widely famous for detecting Tuberculosis. Some studies developed a modified architecture for better accuracy, positively affecting the diagnosis. Vasundhara et al. [115] proposed Deep Learning Normalization – free Network model with 96.91% accuracy, 99.38% AUC, 91.81% sensitivity, and 98.42% specificity on a multi-class classification dataset. Normalization–free–network is created by eliminating normalization layers from ResNets.

Furthermore, it has been demonstrated that computer-aided polyp detection systems utilizing deep learning improve endoscopists’ capacity to identify polyps during routine screening and surveillance colonoscopies. Most of the extra polyps found were tiny and hyperplastic, limiting the study’s therapeutic applicability. However, compared to regular colonoscopy, the computer-aided detection technique produced a considerably higher ADR (29.1% against 20.3%) and a more significant mean number of adenomas found per patient (0.53 versus 0.31). Several CNN architectures have been presented for automated detection in colonoscopic pictures and sequences in the last five years. For example, Mohammed et al. [114] utilized Y-Net-architecture and achieved 85% of accuracy, Misawa et al. [116] proposed a 3D-CNN with 76% of sensitivity, A Faster-R-CNN architecture was used by both Mo et al. [121] and existing architecture utilized by researchers.

However, there might be some challenges to making it possible to replace every outdated or conventionally used technical methodology that is being used in the current healthcare infrastructure because there are certain limitations to the employment of such an advanced technology that would be responsible for taking critical decisions related to the lives of human beings. Overcoming these problems is only possible when each component of the current infrastructure, including the healthcare workers, supports enhancing the capabilities and the current situation. Moreover, it is essential to understand each possible positive and negative aspect of employing such technology that requires a significant number of changes in the currently available infrastructure, which would eventually result in substantial monetary and fiscal support from the governing body of healthcare infrastructure.

AI and ML-based solutions are employable in various sub-fields of healthcare while resolving existing pitfalls in the respective field of study. One such sub-field is diagnostics of numerous widespread diseases and ailments, which cause a continuous increment in the global mortality rate every year. The utilization of solutions based on AI, specifically Deep Learning, can help to resolve this problem to a great extent. Also, at this high population increment rate, it would become difficult for doctors and other workers to complete their jobs with the same efficiency because of overburden and less available time. Therefore, hospitals can resolve this problem with the help of ML and AI-based solutions, which can learn to predict every possible outcome with minimal error at any stage so that further procedures can be accelerated while saving time and other essential resources. Even for hospitals, utilizing a virtual assistant would be economically favorable. Therefore, implementing AI and ML-based solutions for diagnosis purposes can be done without high monetary support. However, it requires large datasets to be prepared to implement such solutions, and hospitals may need to spend some resources to train healthcare workers to use this technology. Therefore, the current infrastructure can digest the involvement of such virtual intelligence with little initial investment in setting up in-house diagnostics centers. Long-run outcomes are expected to balance those early expenses.

Moreover, this situation continues over all possible implementations of AI and ML-based healthcare solutions in all their respective sub-fields of the overall healthcare system, requiring similar efforts from the available infrastructure to solve existing issues and accelerate various procedures to provide faster and better services to patients. Studies show that AI-ML-based solutions can significantly reduce healthcare service costs. However, it might not be economical for the current medical infrastructure to adopt this technology for wide usage and may require significantly high monetary investment, which includes discarding or reducing the use of available resources and setting up requirements for implementing new technology. While analyzing various case studies, which may have yet to be done at the infrastructure level but present good results in their research area, one can draw some important implications related to the employment of AI-based healthcare services at the infrastructure level. Studies show that the cost of many medical procedures can be reduced by up to a significantly high proportion, relieves the patient, and is beneficial for the healthcare service providers. Hospitals can increase their revenues by reducing operational time and the number of resources spent on each patient while attending more patients and utilizing that money for further developments and expansion to provide services with better reach and efficacy.

The following implications would most closely match what these technologies mean for healthcare to resemble the above possibilities of Machine Learning and Artificial Intelligence based interventions in healthcare at the infrastructural level:

- Develop a local or nationwide AI plan for the medical sector, including short- and long-term expectations and objectives, particular projects, finances, and evaluation methods. Establish applicable scenarios to be supported by dedicated financing and opportunities to allow the scalability of AI tools throughout the system; demonstrate that these use cases produce both operational and clinical benefits.
- Set up frameworks for digitization, quality and accuracy, access to data, ownership, risk assessment, privacy and exchange, and system connectivity. Use a mix of financial and performance-based incentives to get people to follow the rules.
- Redefine workforce management and medicinal and pharmaceutical processes to meet the needs of future treatment and AI-centered professionals, and engage up front in developing skills front-line staff and constructing longstanding initiatives for health professionals via ongoing professional growth and diplomas or degrees.
- Offer opportunities and direction for healthcare facilities to cooperate in local or national centers of excellence or innovation clusters.
- Address AI legislation, responsibility, and funding challenges to establish the optimal climate for deploying appropriate, secure, and efficient AI systems while limiting practitioner risk.

Artificial intelligence and Machine Learning based methods can solve many healthcare problems or the most pressing issues, and their advancements offer a slew of intriguing and promising medical applications that can enhance the quality and efficiency of healthcare services. However, significant research efforts are still required in this direction to explore the hidden potential of this technology. Moreover, the requirement for a vast dataset is a significant issue and impediment to making it happen. Despite all the promising technology and machine learning algorithms, we can only achieve the full potential of AI in healthcare if we have enough well-represented data. Digitizing medical records, establishing a uniform data architecture, and creating a secure system to safeguard patient data are all critical steps for the healthcare business. It would be challenging to realize the full potential of AI to improve human health if the healthcare sector had not undergone fundamental transformation and cooperation.

## 5. Conclusions

In this review, we comprehensively analyzed the applications and impacts of utilizing Artificial Intelligence and Machine Learning in healthcare infrastructure. We saw how AI and ML are used in the medical sector and their numerous applications, such as diagnosis, prognosis, research and development, surgeries, administrative tasks, etc. This review also provides a brief history of AI and ML techniques’ evolution and applicability toward strengthening healthcare. It discusses some essential interventions that Artificial Intelligence and Machine Learning have made in this field by enabling machines to behave like humans or intelligently. Some case studies and quantitative analysis have also been included to understand the whole motive from an infrastructural perspective, which provides a clear understanding of the application of such advanced technology from both a patient and hospital point of view. Several studies and many big tech companies have shown how AI and ML can change healthcare completely. However, it would be challenging to utilize this technology globally in the current scenario because it would require a lot of fiscal and monetary support to stabilize it while replacing the existing methodologies. However, with more research and time, it may be possible to reduce healthcare costs and increase the medical sector’s overall strength by exploring these technologies’ potential.

## Figures and Tables

**Table 1:** Summarizing primary advantages of RALP versus, LRP and open.

Perioperative Outcomes	Reduction for Open vs. RALP	Reduction for LRP vs. RALP
Blood loss estimation (mL)	562.5	127.8
Stay length, USA (days)	1.69	0.78
Stay length, non-USA (days)	3.65	1.04

**Table 2:** Findings from the AiCure vs DOT study on Tuberculosis Monitoring for various scenarios captured during the study.

Situation	Cost of AiCure ($ in 2017)	Cost of DOT ($ in 2017)
Base scenario	2668	4894
AI 5% worse ^b^	2860	4894
AI 10% worse ^b^	3011	4894
AI 15% worse ^b^	3164	4894
AI worst case/DOT best case	5278	1607
AI best case/DOT worst case	2883	10,485

^b^ x percent worse means that all monthly closure probabilities within 15 months are reduced by x percentage points, with a base of 0. In the worst/best scenario, the model’s one-way sensitivity parameters are tested at the lower or upper limits of their 95% confidence intervals.

**Table 3:** Summary of sepsis severity prediction study in terms of stay length and mortality rate.

Result	Controlled	Experimental	Reduction (%)
Hospital Stays	13.0 days	10.3 days	20.7
ICU stays	8.40 days	6.31 days	24.88
In-hospital death rate	21.3%	8.96%	12.34

**Table 4:** Advantages and limitations of existing studies employing Artificial Intelligence and Machine Learning in healthcare.

Field of Study	References	Advantages	Limitations and Future Research Directions
Robotic Surgeries	[86,88,92,93,95,97,99,100,101,102,103,104]	Total operational time was reduced by approx. 50%; accuracy of performing minute tasks such as stitching and knotting, improved up to 90%; probability of post-surgery infections, decreased up to 30%; up to 77% blood losses can be prevented; total operational cost reduced up to 35%.	Most of the techniques are designed to achieve maximum precision and do not take patient comfort into attention; the cost of initial setup and maintenance is high; training methodologies for healthcare staff still need to be well explored.
Disease monitoring	[105,108,109,110]	Continuous monitoring can prevent hazardous situations; fine-tuning of on-going treatment is possible; total duration from getting sick to get cured reduces up-to 40%; better treatments can be planned; research and development of medicines can be accelerated.	Existing studies mainly focus on case-wise studies while generalized methodology can be an area of interest for researchers; mixed datasets scenario can be an exciting area as a future work; present study covers most frequent diseases while there is various disease where AI can be utilized; home-based monitoring is not addressed in these studies.
Diagnosis and prognosis	[106,108,111,112,113,114,115,116]	Initial process time can be reduced up to 70%; early prediction can prevent worse conditions; predicted prognosis and expert suggestions can be fused to produce best possible results.	Comparatively high false positive ratio; high miss-classification ratio; extrapolation accuracy still needs to be better than an interpolation; integration with existing technology can be a matter of further studies.
Prediction systems	[107,117,118,119]	Early predictions can help doctors to provide better treatment; intelligent phone-based prediction systems help patients to know their condition without visiting the hospital; can be used to find the root cause of any disease.	High false positive ratio; requires many data for training; high computational power is required for training and prediction; technology is comparatively costly.

## Data Availability

No new data were created.
